# Numerical Absorbing Boundary Conditions Based on a Damped Wave Equation for Pseudospectral Time-Domain Acoustic Simulations

**DOI:** 10.1155/2014/285945

**Published:** 2014-03-11

**Authors:** Carlos Spa, Pedro Reche-López, Erwin Hernández

**Affiliations:** ^1^Mathematics Department, Universidad Técnica Federico Santa María, 2340000 Valparaíso, Chile; ^2^Telecommunication Engineering Department, University of Jaén, Linares, 23700 Jaén, Spain

## Abstract

In the context of wave-like phenomena, Fourier pseudospectral time-domain (PSTD) algorithms are some of the most efficient time-domain numerical methods for engineering applications. One important drawback of these methods is the so-called Gibbs phenomenon. This error can be avoided by using absorbing boundary conditions (ABC) at the end of the simulations. However, there is an important lack of ABC using a PSTD methods on a wave equation. In this paper, we present an ABC model based on a PSTD damped wave equation with an absorption parameter that depends on the position. Some examples of optimum variation profiles are studied analytically and numerically. Finally, the results of this model are also compared to another ABC model based on an hybrid formulation of the scalar perfectly matched layer.

## 1. Introduction

Many real acoustical problems need to be solved through a numerical approach because obtaining an analytical solution is impossible, for example, complex vibroacoustical systems and room impulse responses. Depending on the nature and specifics of the problem itself, it is more feasible to use different particular methods; that is, static problems could be solved by frequency-based methods such as finite [1–3] or boundary elements methods [4, 5], whereas dynamical systems could be solved by time-based methods, for example, the finite difference time domain (FDTD) [6].

In time-based methods, both space and time must be discretized; therefore, a considerable amount of memory is needed to compute the solution. Then, any opportunity to reduce the computational load should be considered under the specifics of the problem. For instance, when an impulse response is the goal, the (pressure) wave equation might be the best equation to solve, instead of the linearized Euler equations, where both pressure and velocity vector are calculated, and intermediate velocities are unnecessarily stored to compute the final pressure impulse response [7].

An interesting and promising numerical method, rarely used in acoustic problems, is the so-called pseudospectral time-domain (PSTD) method, where the spatial derivatives are efficiently computed via discrete Fourier transforms [8]. In multidimensional cases, the computational cost of this method outperforms those based on finite differences. However, to obtain a proper solution, absorbing boundary conditions are needed at the edges of the discrete mesh to avoid the Gibbs phenomenon. Therefore, a perfectly matched layer (PML) is usually included at mesh boundaries, having desirable results as reported in previous works [9, 10].

Although the PSTD technical literature has so far presented the PML for the acoustic Euler equations [11], there exist no solutions for the wave equation. This paper presents an alternative absorbing boundary condition for the wave equation when a PSTD method is used. Toward this goal, a damped wave equation with a spatial dependent damping parameter is presented, where a parameter variation allows controlling the absorption and therefore minimizes those undesirable reflections.

This paper is organized as follows: some basic notions about the PSTD method and a theoretical analysis of the damped wave equation are presented, paying special attention to those cases with a spatially varying damping coefficient. Finally, several experimental cases are presented to demonstrate the proposed method's validity, including a comparison with a previously published method.

## 2. PSTD Methods for the Damped Wave Equation

Recently, new numerical techniques have emerged for solving dynamic problems; one of the most interesting approaches is the PSTD methods [12]. In contrast with the common FDTD methods, PSTD methods are characterized by an isotropic dispersion relation and a less restrictive Courant stability number [8]. So far, they have been successfully applied in many different fields such as acoustic wave propagation [10], piezoelectric transducer modeling [13], or photonic device simulations [14].

In this section, the formulation of the PSTD damped wave equation is presented and the properties of an incrementally progressive damping medium are studied.

### 2.1. Formulation

Assuming that the sound perturbations are infinitesimally smaller than the pressure of the medium and considering the frictional energy losses of the fluid particles, acoustic propagation can be modeled by a scalar acoustic damped wave equation that describes the relationships among the physical quantities of acoustic pressure, density variation, and absorption coefficient of the medium. In a two-dimensional acoustic problem the damped wave equation is of the form:

(1) ∂2p(x,y,t)∂t2+σ∂p(x,y,t)∂t=c2Δp(x,y,t),

where *p*(*x*, *y*, *t*) is the instantaneous acoustic pressure fluctuation of the sound. *σ* ≥ 0 is a damping coefficient that depends on the medium and is expressed in s^−1^, and *c* is the speed of sound propagation in the medium in the absence of damping; that is, *σ* = 0.

To state the method, let us define a 2D Cartesian mesh *Ω*, where the acoustic pressure information, *p*(*x*, *y*, *t*), is only measured at junctions of the grid. Moreover, time variations of the spatial distribution are also captured at certain instants. As a consequence, position and time are transformed to the discrete quantities *x* = *i*Δ*x*, *y* = *j*Δ*y*, and *t* = *n*Δ*t*. Therefore, the quantity defined as the acoustic pressure now becomes its discrete version *p*_*i*Δ*x*,*j*Δ*y*_^*n*Δ*t*^ = *p*_*i*,*j*_^*n*^.

At this point, we are able to use any numerical technique for approximating the partial differential operators of (1). In PSTD, as in the conventional FDTD, we use the well-known first and second order centered finite difference operators for the temporal derivatives,

(2) ∂p(x,y,t)∂t~p|i,jn+1−p|i,jn−12Δt,

(3) ∂2p(x,y,t)∂t2~p|i,jn+1+p|i,jn−1−2p|i,jn(Δt)2.

Both operators have accuracies on the order of *𝒪*(Δ*t*)^2^.

However, for spatial derivatives, we need to make other considerations. From Fourier theory, it is well known that the approximation of the second order spatial derivative [15], for example, in the *x* direction, can be written as

(4) ∂2p(x,y,t)∂x2≈ℱx−1[(ιkx)2ℱx[p(x,y,t)]],

where $\iota =\sqrt{-1}$ is the imaginary unit, *ℱ*_*x*_ and *ℱ*_*x*_^−1^ denote the Fourier transform over the *x*-axis and its invers, respectively, and *k*_*x*_ is the *x* component of the wavenumber.

In the discrete version, we need some additional considerations. We assume a periodic and continuous spatial discrete distribution of the acoustic pressure and we define Δ*x* = Δ*y* and *N*_*μ*_ as the number of grid points in the *μ* direction. We are able to use the discrete Fourier transform (*𝒟ℱ𝒯*) for approximating the spatial derivative at the locations *x* = *i*Δ*x*, *i* = 0,1,…, *N*_*x*_ − 1, which is given by a trigonometric polynomial:

(5) $$\begin{array}{c} \frac{\partial^{2}p\left(x,y,t\right)}{\partial x^{2}}\approx \sum_{m=-N_{x}/2}^{N_{x}/2-1}{\left(\iota k_{x}^{m}\right)}^{2}𝒟ℱ{𝒯}_{x}\left[{p|}_{:,j}^{n}\right]e^{\iota k_{x}^{m}i\Delta x}, \end{array}$$

where *k*_*x*_^*m*^ = 2*πm*/*L*_*x*_, *L*_*x*_ = *N*_*x*_Δ*x* is the length in the *x* direction, and *𝒟ℱ𝒯*_*x*_[*p*|_:,*j*_^*n*^] is the discrete Fourier series defined by

(6) 𝒟ℱ𝒯x[p|:,jn]=1Lx∑i=0Nx−1p|i,jne−ιkxmiΔx,

where the  : symbol denotes all *μ*-coordinates along the straight line cut through the space lattice. Then, the 2D discrete damped wave equation for the Fourier PSTD methods can be easily derived obtaining an expression that reads

(7) p|i,jn+1=σiΔt−2σiΔt+2p|i,jn−1+4σiΔt+2p|i,jn+2(cΔt)2σiΔt+2(𝒟ℱ𝒯x−1[(ιkxm)2𝒟ℱ𝒯x[p|:,jn]]       +𝒟ℱ𝒯y−1[(ιkym)2𝒟ℱ𝒯y[p|i,:n]]).

Note that for *σ*_*i*_ = 0 the PSTD algorithm for the wave equation is recovered. Interestingly, the discrete Fourier transforms in (7) can be obtained efficiently by using a FFT algorithm [16], with a number of operations of the order of (*N*_*x*_log⁡_2_ *N*_*x*_).

Another important feature should be mentioned about this PSTD algorithm: the Courant stability number. This number relates the spatial and temporal sampling constraining the selection of those parameters. For these schemes, it can be proven that

(8) S=cΔtΔx≤2π2,

assuming Δ*x* = Δ*y*.

Finally, it is important to highlight that these algorithms have a less restrictive Courant stability number than the classical FDTD for which the optimum Courant number is $S=1/\sqrt{2}$. By fixing the time discretization and the optimum *S*, in PSTD, we obtain larger spatial samplings; therefore, fewer nodes are required for characterizing the same domain which considerably reduces the computational cost of the algorithms.

### 2.2. Absorbing Boundary Condition Based on an Incrementally Progressive Damping Medium

As indicated in the introduction, the scope of this paper is to establish the damped wave equation as ABC method for the aforementioned PSTD methods; for this reason, this section provides a brief background needed to understand the main features of the physical model of an incrementally progressive damping medium.

The acoustic wave propagation characteristics in any medium are defined by means of the intrinsic impedance, *η*, and the propagation constant *γ* [17]. It can be shown that the propagation constant is a complex quantity of the form *γ* = *α* + *ιβ*, where the real part of the propagation constant is the attenuation constant *α* = *ℜ*(*γ*) in nepers per meter and the imaginary part is the phase constant *β* = *ℑ*(*γ*) expressed in radians per meter. The attenuation constant causes an exponential decrease in the fields' amplitude along the propagation direction through the medium. The phase constant represents the change in phase per distance along the wave path traveled at any instant. Note that this quantity is strongly related to the angular frequency *ω*. For example, if *α* = 0 and *β* = *ω*/*c*, the propagation constant, *γ*, is purely imaginary and is referring to an undamped medium, which indicates a lossless medium that does not absorb the acoustic field. Nevertheless, in general, the propagation constant has a complex magnitude depending on *σ* according to $\gamma =(\iota \omega /c)\sqrt{1-\iota \tan\delta }$, where tan*δ* = *σ*/*ω* is the acoustic loss tangent.

Otherwise, the intrinsic (or characteristic) impedance is defined as the relation between the complex amplitudes of the pressure and velocity when a plane wave is propagating through a medium. Like the propagation constant, the intrinsic impedance obtained is a complex number that depends on *σ* (i.e., tan*δ*), $\eta =\rho c/\sqrt{1-\iota \tan\delta }$. Note that for *σ* = 0 the real impedance *η* = *ρc* is achieved, which is the intrinsic impedance of the undamped medium with density *ρ* and velocity *c*.

**Figure 1:**
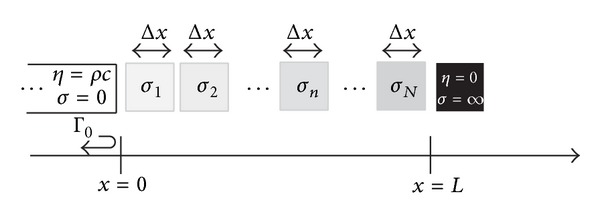
Normal wave propagation through *N* layers with increasing damping coefficient.

Now, we will address the 1D problem of a plane wave moving from a lossless medium with intrinsic impedance *η*_0_ = *ρc* to an absorbing region of thickness *L*. This region has the peculiarity of being composed of multiple layers with damping coefficients that progressively increase. At the end of the domain, a sound soft boundary condition is defined (*η*_*N*+1_ = 0 or *σ*_*N*+1_ = *∞*). The absorbing region is represented in Figure 1. The region can be viewed as being made up of *N* layers of thickness Δ*x*, with a damping coefficient *σ*_*i*_, *i* = 1,2,…*N*. The overall reflection response, Γ_0_, can be obtained in several ways [18], such as by the impedance propagation at the interfaces, by the propagation of the reflection responses, or by transmission matrices obtaining the following recurrence relation:

(9) ΓN=ηN+1−ηNηN+1+ηN,

(10) Γi−1=((ηi−ηi−1)/(ηi+ηi−1))+Γie−2γiΔxi1+((ηi−ηi−1)/(ηi+ηi−1))Γie−2γiΔxi.

Note that the reflection coefficients Γ_*i*_ are obtained in reverse order until Γ_0_ is computed.

It is worth mentioning that the characteristic impedance, *η*_*i*_, and the propagation constant *γ*_*i*_ are functions of the damping coefficient. Likewise, the damping coefficient can be written as a function of position. Hence, depending on the variation of *σ*_*i*_, the total reflection coefficient, Γ_0_, will be directly affected. Therefore, it is important to emphasize that the profile chosen for smoothly varying the damping coefficient will affect the total reflection coefficient obtained.

To illustrate this theory based on an incrementally progressive damping medium, we present an analytical study choosing different variation profiles based on a potential variation of the form

(11) $$\begin{array}{c} \sigma_{i}=\sigma_{\max}{\left(\frac{i\Delta x}{L_{\text{abc}}}\right)}^{m}, \end{array}$$

where *L*_abc_ is the length of the ABC. The main objective of this is to assess for which *m* value we obtain the lowest reflection coefficients. With this objective, a simple experiment is designed. We define a semi-infinite 1D domain of a nondamped medium, where at the end of it a transition region is defined by using the potential profile; see (11). For evaluation purposes, the magnitude of the total reflection coefficient will be shown in the specific case, where *σ*_max⁡_ = 16000 s^−1^ and the thickness of the absorbing region is *L* = 0.54 m. Under these conditions, the magnitude of the reflection coefficient, (9) and (10), was calculated for frequencies *f* ≤ 4 kHz.

**Figure 2:**
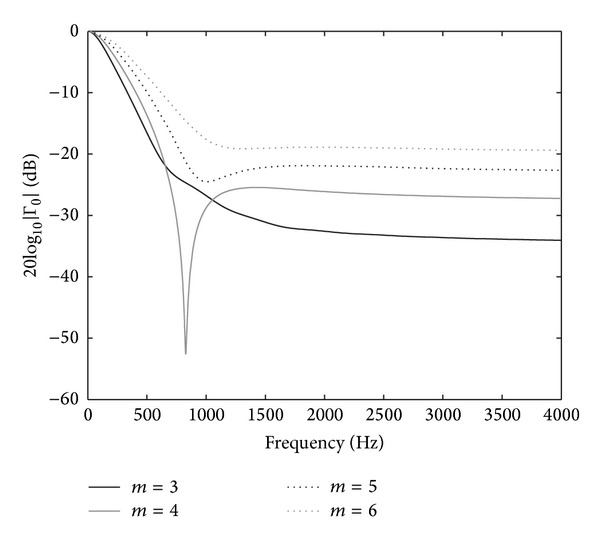
The reflection coefficient magnitude using different potential damping coefficient profiles (*m* = {3,4, 5,6}).

In Figure 2, we plot the magnitude of the reflection coefficient |Γ_0_| versus the frequency by using different potential profiles (11). It is worth noting that we only show the results obtained for *m* = 3, 4, 5, and 6; however, we tested the profile covering a large range of *m*. Regardless of the profile chosen, we observe the worst results in the low frequency range (frequencies from 0 to 600 Hz), where the reflection coefficient amplitude values are decreased from 0 to −20 dB in all the cases. Conversely, in the rest of the frequency range (*f* > 1 kHz) the magnitude of the reflection coefficient remains almost constant, with values lower than −20 dB except for *m* = 6, where it has values slightly higher than −20 dB.

Although the shape of the results is very similar in terms of |Γ_0_|, we observe the best results for *m* = 3 and 4. In both cases and for frequencies *f* > 900 Hz, we obtain almost constant reflection coefficient values, which are around −30 dB. These coefficients are 10 dB lower than those obtained for *m* = 5 or 6. Moreover, for *m* = 4 and in a frequency range around 800 Hz, a peak of maximum absorption appears obtaining values of |Γ_0_| of the order of −50 dB. Note that this peak is strongly related to the length of the transition region.

It is worth emphasizing that these results are completely expected because the reflection coefficient has a strong dependence on the frequency. As observed in (9) and (10), the reflection coefficient depends directly on the propagation constant which is inversely proportional to the frequency. Thus, for low frequency values we obtain large values for the reflection coefficient.

Apart from this feature of the damped wave equation, we observe reasonable absorptions in over nearly the whole range of frequencies. Thus, it seems reasonable to consider this strategy of defining incrementally progressive damping medium to obtain high absorptions of the incident waves.

## 3. Numerical Analysis

The next step consists of validating this theory in a two-dimensional numerical experiment. In this case, more complex phenomena arise because the oblique incidence and the numerical dispersion error must be considered. In this section, we try the two best potential profiles obtained in 1D based on this theory and compare the different proposals through objective and quantitative measurements. We extend the 1D analysis of the specific potential profiles via 2D simulations providing a frequency-angular analysis of the reflection coefficient produced by a transition region. Furthermore, to obtain a comparative analysis of the method, we present another ABCs based on PML for the scalar wave equation, but in this case, combined with a PSTD algorithm. Finally, results of both methods are analyzed and compared in the discussion.

### 3.1. Experimental Setup and Results

Because the study presented in Section 2.2 is developed in one dimension, it is necessary to extend this work to 2D, to observe the angular dependence of the reflection coefficient due to the absorption coefficient variation in the media.

The numerical setup has been inspired by [19] for FDTD [20] methods. The experimental setup consists of a two-dimensional rectangular domain with a height and weight of 750 × 1500 nodes. In all simulations performed, we fixed the frequency sampling *f*_*s*_ = 16 kHz and the maximum Courant stability number, $S=2/\pi \sqrt{2}$. Under these assumptions, a volumetric acoustic source is emitted located at nodes (*i*_*s*_, *j*_*s*_) = (718 + *k*, 750 + *l*), for *k*, *l* = {0,1}, that emits a windowed sinc function with a bandwidth of 4 kHz. We also define a transition region that goes from *i*_1_ = 735 to *i* = *i*_1_ + *N*, where *N* = 15. In this transition region and for the damped wave equation, we vary the absorption coefficient *σ*_*i*_ from *σ*_1_ to *σ*_*N*_.

According to the 1D case, a domain has been defined that contains subdomains with different absorption coefficients. The idea is that if transitions between media are sufficiently smooth, the reflection coefficient due to the changing media will be low enough to consider the damped equation proper ABC. As in the 1D experiment of Section 2.2, we focus on the potential variation of the damping coefficient; see (11), for the specific case of *m* = 3 and 4.

The numerical reflection coefficient, Γ_num_, is obtained at different receivers located at (*i*_*s*_, *j*_*s*_ + *l*) for 0 ≤ *l* ≤ 250 providing an angular study of the absorbing behavior. Therefore, the goal of this experiment is to numerically measure the magnitude of the reflection coefficient as a function of the angle of incidence (determined by the receiver positions) and the frequency. Finally, note that the domain boundaries are fixed to 0 and the simulations were run for 1024 time steps to guarantee that there were no rebounds due to the soft walls of the boundaries.

It is important to highlight that, for this 2D numerical study, we require a more complex analysis because features such as oblique incidence or dispersion error radically affect the values of the reflection coefficient. Hence, to objectively measure the profiles, we consider different parameters to get an idea regarding the reability of this approach. For example, one of the parameters we propose as figure of merit (FoM) is the value of the Bode-Fano integral in the bandwidth of interest. The Bode-Fano integral has been proposed as an objective tool for assessing the bandwidth of antennas and other circuits [21, 22]. Consider

(12) $$\begin{array}{c} \text{FoM}=\int_{0}^{f_{\max}}\log_{10}\left(\frac{1}{\left|\Gamma_{\text{num}}\left(f\right)\right|}\right)\text{d}f. \end{array}$$

Larger values of this figure of merit imply better performances and less reflected energy at the beginning of the ABC region in the bandwidth of interest [0, *f*_max⁡_].

**Figure 3:**
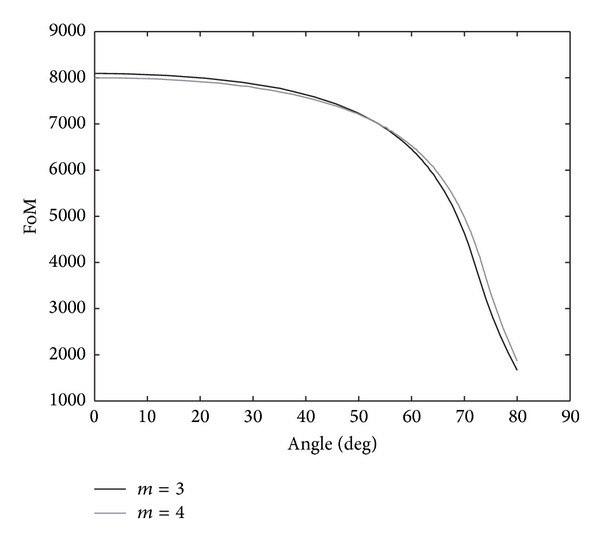
FoM versus angle using potential profiles *m* = 3 and *m* = 4.

In Figure 3 we show the results of the FoM versus angle derived from the 2D simulations of the PSTD damped wave equation. It is important to highlight that, as in the 1D case, we only show the best performances that were obtained for *m* = {3,4} and *σ*_*N*_ = 16000 s^−1^. From the values of the FoM, we conclude that independent of the *m* chosen the data are quite similar. For example, one common feature observed in all simulations is that the magnitude of the FoM decreases when the angle becomes large. For a wide range of angles, from approximately 0 to 55 degrees, the values of the FoM remain constant at approximately 8000 s^−1^. Conversely, this quantity is radically decreased at other angles and attains values on the order of 2000–2500 s^−1^. Therefore, the absorption behavior of the damped wave equation is strongly affected by the incidence angle.

**Figure 4:**
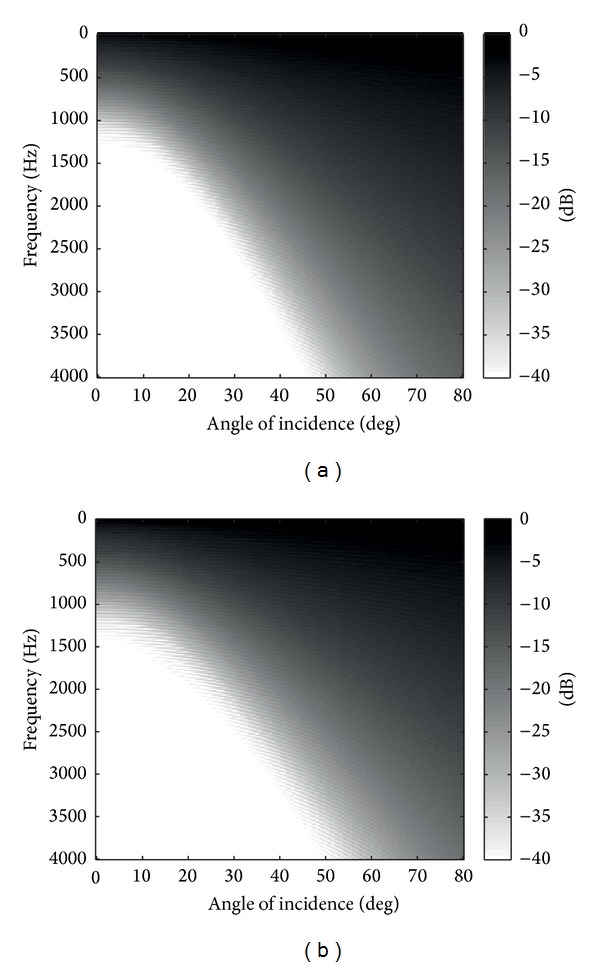
The reflection coefficient |Γ_num_| in decibels as a function of the angle and frequency obtained with the numerical PSTD scheme, (7). (a) for *m* = 3 and (b) for *m* = 4.

On the other hand, we depicted the values of the reflection coefficient magnitude in Figure 4, again, for potential profiles *m* = 3 and 4. The plots correspond to the reflection coefficient as a function of the angle of incidence, *θ*, and the frequency. The magnitude of the reflection coefficient is plotted on a graded scale, where black corresponds to a few negative dBs and white corresponds to |Γ_num_| less than −40 dB. It can be seen that both plots are very similar. The best results are obtained for angles *θ* < 20. Over nearly the whole frequency range, 600 < *f* < 4000 Hz, the reflection coefficients obtained are less than −35 dB on a logarithmic scale. In other words, this value in dB corresponds to a reflection coefficient on the order of approximately ~1.8%, which are values that are sufficiently small to indicate almost perfectly absorbing boundary conditions. On the other hand, higher reflections are obtained in the lowest frequency range (from 20 to 600 Hz). These results are completely expected because the intrinsic impedance and the propagation constant of media have a strong dependence on the frequency. Otherwise, oblique incidence appears to worsen the results. It is observed from the figure that, for *θ* > 20, the frequency range with reflections lower than −35 dB is decreased linearly with the angle. Nevertheless, there is a relevant region where the reflection coefficient remains low enough, of the order on −20 dB, to be considered acceptable absorption values.

**Figure 5:**
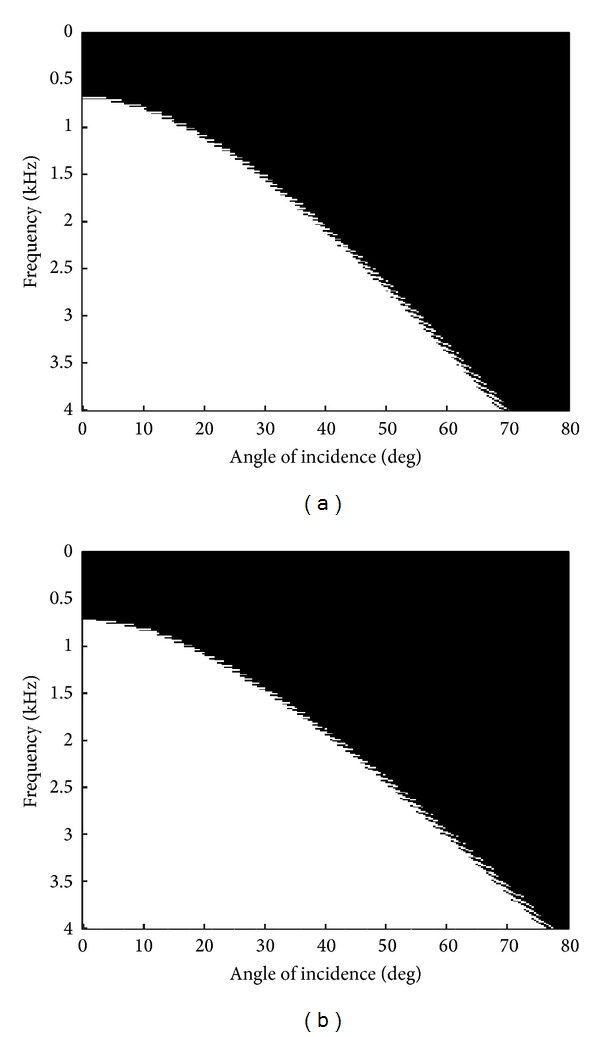
BW_20_ as a function of the angle and the frequency obtained with the numerical PSTD scheme, (7). (a) For *m* = 3 and (b) for *m* = 4.

In fact, it is necessary to define more parameters to determine which is the acceptable region or the effective region of the method. For example, another tool that we propose for assessing the performance of a specific damping profile is the useful bandwidth. We define the useful bandwidth BW_20_ as the range of frequencies where the magnitude of the reflection coefficient at the receivers is less than −20 dB. In Figure 5, we plot the BW_20_ for *m* = 3 and *m* = 4. The white reflection coefficients are equal to or lower than −20 dB whereas black indicates greater reflections. As shown, the results in each case are very similar. The figures clearly show that the useful bandwidth decreases linearly when the angle increases. Note that slightly better results for large angles are obtained when *m* = 4. However, we conclude that the results are almost equal in both cases.

### 3.2. A Comparative Method: The Scalar Perfectly Matched Layers

Although the analysis of Eulerian PML for PSTD methods has been already treated, surprisingly, according to these authors, in the context of the ABC implementation for the discrete wave equation, there are only contributions based on FDTD approximations; see [23–26].

In this section, we present the acoustic version of the scalar PML [27] when a Cartesian uniform rectangular mesh is used. The discrete update equations of scalar PML are obtained by using centered finite difference operators of second order accuracy for temporal derivatives and combining forward/backward finite difference operators for spatial derivatives. At the end, we obtain the following numerical scheme:

(13) D1|i+1/2,jn+1/2=(1−φxiρc2Δt/21+φxiρc2Δt/2)D1|i+1/2,jn−1/2      +ΔtΔx(1+φxiρc2Δt/2)(p|i+1,jn−p|i,jn),D2|i,jn+1/2=(1−φxiρc2Δt/21+φxiρc2Δt/2)D2|i,jn−1/2      +D1|i+1/2,jn+1/2−D1|i−1/2,jn+1/2Δx(1+φxiρc2Δt/2)      −D1|i+1/2,jn−1/2−D1|i−1/2,jn−1/2Δx(1+φxiρc2Δt/2),p|i,jn+1=p|i,jn−1+2p|i,jn     +c2Δt(D2|i,jn+1/2−D2|i,jn−1/2)     +S2(p|i,j+1n+p|i,j−1n−2p|i,jn),

where *D*_1_ is referring to the normal component of the velocity and *D*_2_ is proportional to the acoustic pressure. Note that *ρ* represents the density of the propagation medium and *φ*_*x*_^*i*^ is the PML absorption coefficient in the *x* direction.

For this type of ABC, the strategy of defining a transition region that goes from 0 to *φ*_*x*_^*N*_PML_^ with a smooth variation is also used. As reported in [28], to obtain the optimum absorption and to minimize the numerical reflection due to abrupt changes of media, *φ*_*x*_^*i*^ should vary as follows:

(14) $$\begin{array}{c} \varphi_{x}^{i}=-{\left(\frac{i}{N_{\text{PML}}}\right)}^{q}\frac{\left(q+1\right)\log_{10}R_{0}}{\rho cN_{\text{PML}}\Delta x}, \end{array}$$

where *N*_PML_ refers to the total number of PML modes in *x* direction and  *q* and *R*_0_ are two constants that are 5 and 10^−2^, respectively.

One important drawback of these numerical ABC is that they cannot be extended to PSTD formulations, because it is not possible to use Fourier techniques for computing the spatial derivative of the quantity *D*_1_. One of the main problems of using Fourier techniques for computing spatial derivatives is that continuous periodic spatial distributions are assumed. In this case, it is impossible to guarantee continuity to the normal component of the velocity. Therefore, the Gibbs phenomenon would appear every time we compute the spatial derivative of *D*_1_.

Therefore, in order to use these ABC in a PSTD simulation, we implement the PSTD algorithm for the scalar wave equation in the region, where *σ*_*i*_ = 0, whereas in the absorbing region, we use the PML equation (see (13)) formulated in FDTD. Therefore, in the end, we obtain a hybrid algorithm that combines two different methods. As well as in the damped simulations, we define a transition PML region of 16 nodes and we preserve in the whole domain the stability number to $S=2/\pi \sqrt{2}$ which is lower than the maximum stability number for FDTD, $S=1/\sqrt{2}$. Otherwise, the simulations would be completely unstable.

### 3.3. Discussion

As mentioned, to obtain a comparative analysis of the method and better understand these results, we carry out the same experiment using PML for the scalar wave (13). Similarly, we define the same transition region for PML but in this case we vary *φ*_*x*_^*i*^ from *φ*_*x*_^1^ to *φ*_*x*_^*N*_PML_^ according to the variation profile given by (14).

**Figure 6:**
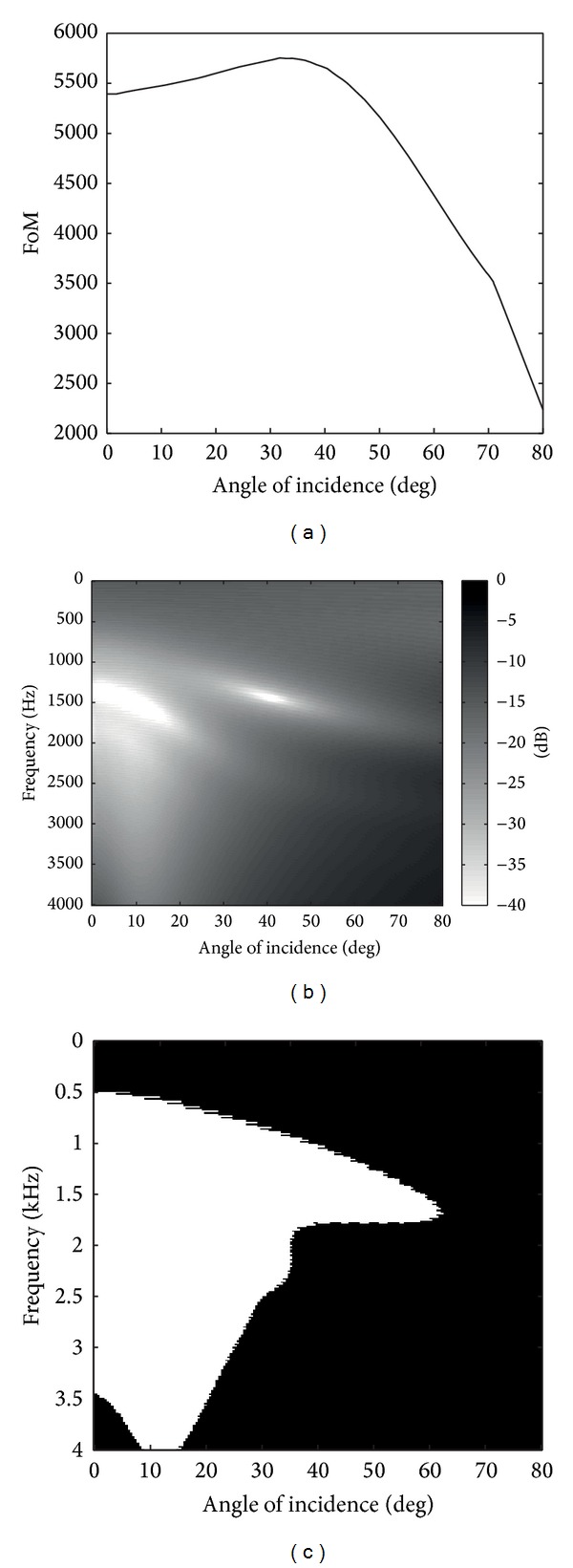
(a) The FoM of the PML is depicted with the optimum profile equation (14). (b) In this gray scale plot we show the reflection coefficient obtained in the PML simulation versus angle and frequency. (c) The BW_20_ is plotted again versus angle and frequency.

In Figure 6(a), we present the results of the FoM. The data were obtained under the same numerical assumptions than in the experiment described in Section 3.1. In this case, the worst results are obtained at large angles, whereas the best results are obtained at approximately 40 degrees. Note that, independent of the angle, we obtain lower values of the FoM compared to the results obtained from the PSTD damped wave equation, see Figure 3. It shows that the PSTD damped wave equation absorbs more of acoustic field than does the hybrid formulation of PML.

As for the damped wave equation, in Figures 6(b) and 6(c), we again depict the reflection coefficient and the BW_20_ as functions of the frequency and the angle. Both plots show worst results than those obtained with the damped wave equation, Figures 4 and 5, over nearly the whole angular-frequency range. One remarkable feature that is derived from these results is that the reflection coefficient seems more isotropic and independent of the frequency than our proposal. For example, in the low frequency range, although both strategies have values greater than −20 dB, the PML simulations give better results than the damped version. This is one of the most representative features of the PML because it is well known that these ABC are defined to have absorptions independent of the frequency. The problem that we have in PSTD is that we obtain higher reflections than expected due to the hybrid formulation of the scheme.

## 4. Conclusions

There are a constantly growing number of applications where the use of a time-based numerical approach for the acoustic wave equation is needed, such as architectural acoustic and music synthesis. Among them, the boundary conditions are a permanent and challenging research area for computational acousticians. However, there is an important lack of research on absorbing boundary conditions for the wave equation. In fact, the most popular absorbing boundary condition, the so-called* perfectly matched layer,* has been always developed to be used on Euler equations, but very few attempts to use them on the wave equation can be found in the PSTD technical literature.

Towards that end, this paper is devoted to a new proposed method based on a damped wave equation and a spatial-dependent damping parameter profile to reduce the wave energy on the nearby boundaries. In this proposal, the Fourier pseudospectral time-domain is used, because of its reduced computational cost compared to other time-domain classical methods. Moreover, the mandatory use of absorbing boundary conditions to avoid the so-called Gibbs effect makes this choice interesting. However, it is worth noting that this formulation can be straightforwardly extended to any other variation of the PSTD methods such as Chebyshev [11] or *K*-space [29] approximation.

One of the difficulties of using this proposal lies in choosing an appropriate variational damping profile. In this regard, a figure of merit model is presented, to help select a profile. Moreover, to demonstrate the reliability of this new model, this model is compared to a wave equation-based PML absorbing boundary condition in a 2D mesh. Based on these results, it performs better at high frequencies for a higher range of incident angles with absorption below −20 dB. For the same range of angles, both methods have similar behavior at low frequencies. Finally, it should be noted that this new proposal may provide a starting point in the research of new damping parameter distribution profiles that are able to produce better results.
